# Supplementary material for: Clinical Characterization of Respiratory Syncytial Virus Infection in Adults: A Neglected Disease?
Source: Viruses. 2023 Aug 31;15(9):1848. doi: 10.3390/v15091848 (PMC10536488; doi:10.3390/v15091848)
Supplement: Supplementary file 1 [file viruses-15-01848-s001.zip › viruses-2531526-supplementary.pdf]

Supplementary Tables

Table S1. General characteristics of each group in the respiratory surveillance cohort, by infectious agent.

	FluA					FluB					RSV				
	**N response**	**N positive or mean**	**% or s.d.**	**p-value**	**X^2; df**	**N response**	**N positive or mean**	**% or s.d.**	**p-value**	**X^2; df**	**N response**	**N positive or mean**	**% or s.d.**	**p-value**	**X^2; df**
**Age group**															
Adult	36	30	83.33%	**<0.001**	16; 1	9	7	77.78%	0.096	2.778; 1	51	23	45.10%	0.484	0.490; 1
Elderly	36	6	16.67%			9	2	22.22%			51	28	54.90%		
Mean age	36	38.56	19.51	NA	NA	9	42.89	18.19	NA	NA	51	59.20	18.21	NA	NA
**Sex**															
Female	36	14	38.89%	0.182	1.778; 1	9	7	77.78%	0.096	2.778; 1	51	26	50.98%	0.889	0.020; 1
Male	36	22	61.11%			9	2	22.22%			51	25	49.02%		
**Ethnicity**															
White	33	29	87.88%	**<0.001**	44.364; 2	8	8	100.00%	NA	NA	51	46	90.20%	**<0.001**	74.235; 2
Black	33	3	9.09%			8	0	0.00%			51	3	5.88%		
Mixed race	33	1	3.03%			8	0	0.00%			51	2	3.92%		
**Education**															
Primary incomplete	31	6	19.35%	0.250	2.774; 2	7	1	14.29%	0.565	1.143; 2	46	26	56.52%	**0.004**	11.261; 2
Primary	31	0	0.00%			7	0	0.00%			46	0	0.00%		
High school	31	12	38.71%			7	3	42.86%			46	9	19.57%		
College	31	13	41.94%			7	3	42.86%			46	11	23.91%		
**Comorbidities**	33	13	39.39%	0.223	1.485; 1	5	2	40.00%	0.655	0.200; 1	46	39	84.78%	**<0.001**	22.261; 1

Table S2. Clinical characteristics of patients infected by influenza A and B and respiratory syncytial virus in the respiratory surveillance cohort, December 2021–April 2022.

	Influenza A			Influenza B			RSV			
	**N response**	**N positive or mean**	**% or s.d.**	**N response**	**N positive or mean**	**% or s.d.**	**N response**	**N positive or mean**	**% or s.d.**	**p-value**
**Sex**										
Female	30	11	36.67%	7	6	85.71%	23	11	47.83%	0.064
Male	30	19	63.33%	7	1	14.29%	23	12	52.17%	
**Ethnicity**										
White	27	23	85.19%	7	7	100.00%	23	19	82.61%	0.739
Black	27	3	11.11%	7	0	0.00%	23	2	8.70%	
Mixed race	27	1	3.70%	7	0	0.00%	23	2	8.70%	
**Education**										
Primary incomplete	25	3	12.00%	5	0	0.00%	20	8	40.00%	0.127
Primary	25	0	0.00%	5	0	0.00%	20	0	0.00%	
High school	25	11	44.00%	5	2	40.00%	20	7	35.00%	
College	25	11	44.00%	5	3	60.00%	20	5	25.00%	
**Comorbidities**	28	8	28.57%	5	2	40.00%	20	13	65.00%	**0.042**
Cardiopathy	28	0	0.00%	5	0	0.00%	20	3	15.00%	0.073
Blood pressure	28	3	10.71%	5	2	40.00%	20	5	25.00%	0.205
Hematological disease	28	0	0.00%	5	0	0.00%	20	1	5.00%	0.431
Down syndrome	28	0	0.00%	5	0	0.00%	20	2	10.00%	0.180
Liver disease	28	0	0.00%	5	0	0.00%	20	4	20.00%	**0.028**
Asthma	28	0	0.00%	5	0	0.00%	20	0	0.00%	NA
Diabetes	28	1	3.57%	5	0	0.00%	20	5	25.00%	0.049

Neurovascular disease	28	0	0.00%	5	0	0.00%	20	1	5.00%	0.431
Neurological disease	28	0	0.00%	5	0	0.00%	20	0	0.00%	NA
Pneumopathy	28	0	0.00%	5	0	0.00%	20	2	10.00%	0.180
Immunosuppression	28	0	0.00%	5	0	0.00%	20	1	5.00%	0.431
Kidney disease	28	0	0.00%	5	0	0.00%	20	3	15.00%	0.073
Obesity	28	1	3.57%	5	0	0.00%	20	1	5.00%	0.868
Smoker	25	4	16.00%	3	0	0.00%	17	3	17.65%	0.736
Cancer	28	2	7.14%	5	1	20.00%	20	1	5.00%	0.521
**Signs and symptoms**										
Fever	28	19	67.86%	4	1	25.00%	19	5	26.32%	**0.012**
Headache	28	16	57.14%	4	1	25.00%	19	3	15.79%	**0.014**
Myalgia	28	18	64.29%	4	0	0.00%	19	3	15.79%	**<0.001**
Nasal congestion	28	16	57.14%	4	3	75.00%	19	5	26.32%	0.058
Cough	28	22	78.57%	4	2	50.00%	19	17	89.47%	0.183
Sore throat	28	11	39.29%	4	2	50.00%	19	3	15.79%	0.165
Dyspnea	28	2	7.14%	4	0	0.00%	19	8	42.11%	**0.007**
Respiratory distress	28	0	0.00%	4	0	0.00%	19	6	31.58%	**0.003**
Diarrhea	28	1	3.57%	4	0	0.00%	19	4	21.05%	0.112
Vomiting	28	0	0.00%	4	0	0.00%	19	0	0.00%	NA
Abdominal pain	28	2	7.14%	4	1	25.00%	19	1	5.26%	0.402
Malaise	28	6	21.43%	4	0	0.00%	19	4	21.05%	0.589
Loss of smell	28	1	3.57%	4	0	0.00%	19	0	0.00%	0.658
Loss of taste	28	1	3.57%	4	0	0.00%	19	0	0.00%	0.658
**Hospitalization**	30	30	100.00%	7	7	100.00%	23	23	100.00%	NA
**ICU**	29	0	0.00%	7	1	14.29%	23	3	13.04%	0.125
**Ventilatory support**	29	1	3.45%	7	1	14.29%	23	8	34.78%	**0.011**
**Death**	29	3	10.34%	7	3	42.86%	20	8	40.00%	**0.032**
